# Supplementary material for: A quasi-solid-state high-rate lithium sulfur positive electrode incorporating Li10GeP2S12
Source: Commun Mater. 2025 Aug 5;6(1):175. doi: 10.1038/s43246-025-00901-4 (PMC12325082; doi:10.1038/s43246-025-00901-4)
Supplement: Supplementary file 2 — A Quasi-Solid-State High-Rate Lithium sulfur Positive Electrode incorporating Li10GeP2S12 Supplementary Information [file 43246_2025_901_MOESM2_ESM.pdf]

A Quasi-Solid-State High-Rate Lithium Sulfur Positive Electrode incorporating $Li_{10}GeP_2S_{12}$

Boyi Pang[1,2], Huanxin Li[1], Yiming Guo[2,3], Bochen Li[3], Feiran Li[4], Huw Parks[1,2], Liam Bird[2,5], Thomas S. Miller[2,3], Paul R. Shearing[2,5], Rhodri Jervis[2,3], James B. Robinson[1,2*]

[1]Advanced Propulsion Lab (APL), University College London, Marshgate, London, E20 2AE, UK
[2]Faraday Institution, Quad One, Becquerel Avenue, Harwell Science and Innovation Campus, Didcot, OX11 0RA, UK
[3]Electrochemial Innovation Lab (EIL), University College London, Gower Street, London, WC1E 6BT, UK
[4]Imperial College London, South Kensington Campus, London, SW7 2AZ, UK
[5]ZERO institute, University of Oxford, Wellington Square, Oxford OX1 2JD, UK

*Author to whom correspondence should be addressed: j.b.robinson@ucl.ac.uk

Supplementary Information

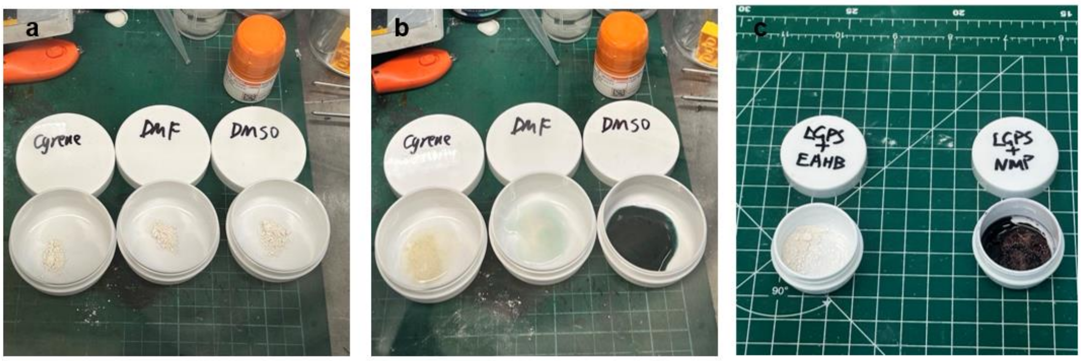

Figure S1. Photographs of LGPS with different solvents: a: LGPS; b: LGPS with dihydrolevoglucosenone (Cyrene), dimethylformamide (DMF), dimethyl Sulfoxide (DMSO); c: LGPS with ethyl acetate (EA) & hexyl butyrate (HB) and n-methyl-2-pyrrolidone (NMP) after drying;

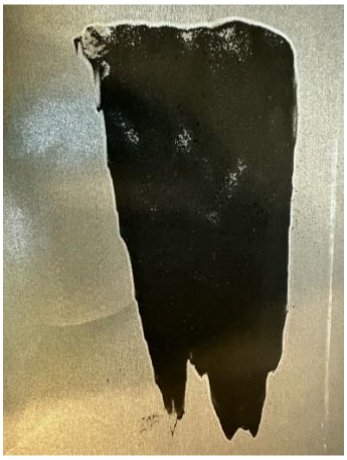

Figure S2. Photograph of cathode material after coating and drying when PVDF-HFP is used as binder and EA is used as solvent

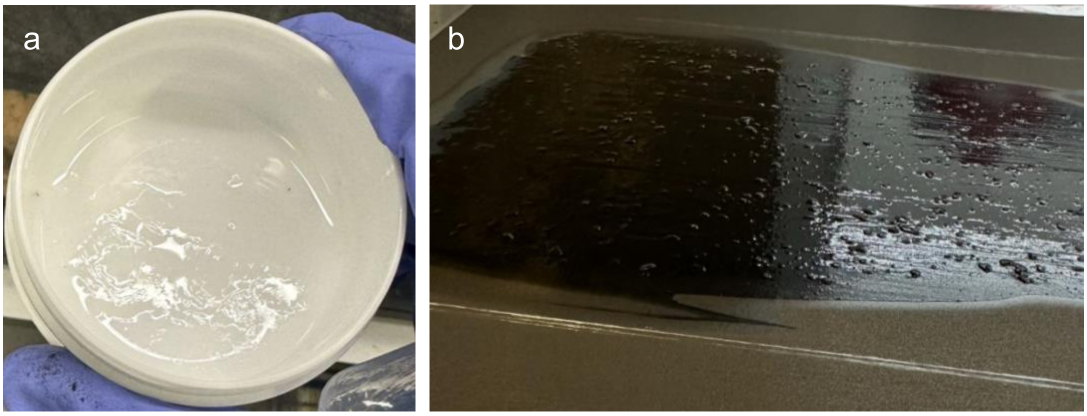

Figure S3. Photographs of PVDF-HFP&EA solution drops in cold HB solution. a) Jelly colloids in solution. b) Jelly colloids after coating.

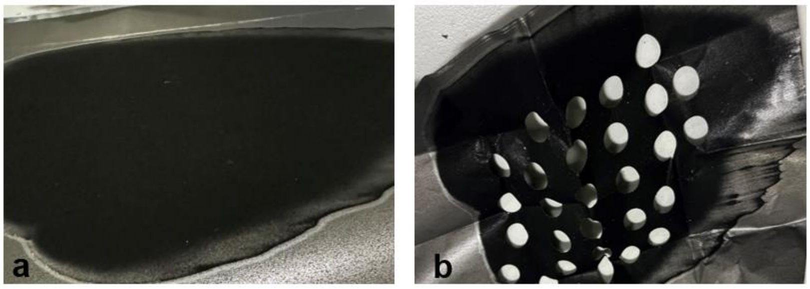

Figure S4. Photographs of coating of PVDF-HFP with EA&HB: a: Coating after drying; b: Coating after cutting and folding.

Table S1. Values and errors of fitted circuit elements for coin cells assembled from KB and KB+LGPS electrodes

Name / Value (relative error)	Resistance 1	Resistance 2	CPE Q 1	CPE Alpha 1	Resistance 3	CPE Q 2	CPE Alpha 2	CPE 3	Warburg Coeff 1
KB	1.39	91.51	1.01E-5 (1%)	0.76474	N.A.	0.023728	1 （1.2%）	N.A.	3.68 (6.3%)

	(0.27%)	(0.36%)		(0.18%)		（7.2%）			
KB+LGPS	1.99	43.7	1.049E-5	0.75314	1.07	0.023648	1	0.009708	11.5
	(2%)	(0.46%)	(0.8%)	(0.25%)	(4%)	(13%)	(6.4%)	(7.1%)	(11%)

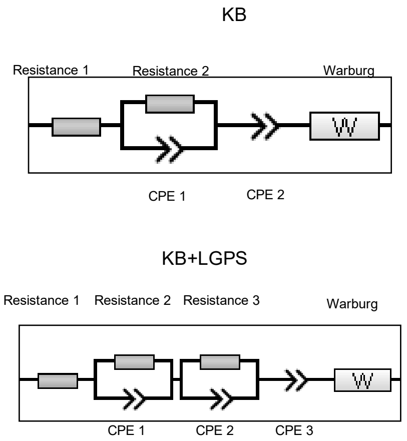

Figure S5. EIS and equivalent circuit model of fresh coin cells;

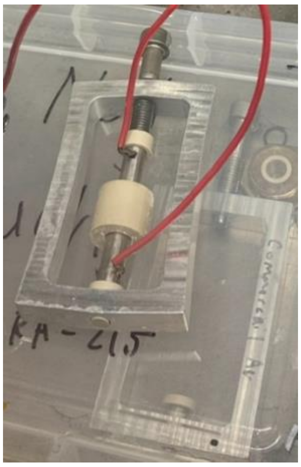

Figure S6. Photographs of LGPS powder testing device for EIS
